# Adversarial Defense Method Based on Latent Representation Guidance for Remote Sensing Image Scene Classification

**DOI:** 10.3390/e25091306

**Published:** 2023-09-07

**Authors:** Qingan Da, Guoyin Zhang, Wenshan Wang, Yingnan Zhao, Dan Lu, Sizhao Li, Dapeng Lang

**Affiliations:** College of Computer Science and Technology, Harbin Engineering University, Harbin 150001, China; da_qing_an@hrbeu.edu.cn (Q.D.); zhangguoyin@hrbeu.edu.cn (G.Z.); wangwenshan@hrbeu.edu.cn (W.W.); zhaoyingnan@hrbeu.edu.cn (Y.Z.); sizhao.li@hrbeu.edu.cn (S.L.); langdapeng@hrbeu.edu.cn (D.L.)

**Keywords:** adversarial denoising, self-supervised learning, latent representation, normalized mutual information, cross-entropy

## Abstract

Deep neural networks have made great achievements in remote sensing image analyses; however, previous studies have shown that deep neural networks exhibit incredible vulnerability to adversarial examples, which raises concerns about regional safety and production safety. In this paper, we propose an adversarial denoising method based on latent representation guidance for remote sensing image scene classification. In the training phase, we train a variational autoencoder to reconstruct the data using only the clean dataset. At test time, we first calculate the normalized mutual information between the reconstructed image using the variational autoencoder and the reference image as denoised by a discrete cosine transform. The reconstructed image is selectively utilized according to the result of the image quality assessment. Then, the latent representation of the current image is iteratively updated according to the reconstruction loss so as to gradually eliminate the influence of adversarial noise. Because the training of the denoiser only involves clean data, the proposed method is more robust against unknown adversarial noise. Experimental results on the scene classification dataset show the effectiveness of the proposed method. Furthermore, the method achieves better robust accuracy compared with state-of-the-art adversarial defense methods in image classification tasks.

## 1. Introduction

The development of deep learning has led to a revolution in remote sensing image (RSI) analysis. With their excellent feature extraction capabilities and end-to-end training mode, deep neural networks can provide more accurate and efficient solutions for environmental monitoring [1], land use classification [2], object detection [3], and other application fields. However, recent studies have shown that deep neural networks are vulnerable to adversarial examples [4], which can mislead or even induce the model’s predictive behavior through embedded adversarial noise. Normally, adversarial noise does not cause changes in human perception; however, they can easily attack the intelligent systems that humans rely upon [5]. Recently, the study of adversarial examples has been extended to the field of RSI analysis [6,7,8]. For examples, a well-camouflaged drone [9] may be recognized as a bird by an intelligent system, and a military installation with an adversarial patch may avoid aerial detection [10]. Obviously, the existence of adversarial examples carries hidden dangers in military applications and in other fields that have high security requirements [11]. For this reason, it is of great significance to carry out research on adversarial noise defense methods based on the scene classification of RSIs.

Adversarial training (AT) is considered to be a very effective method of adversarial defense [12,13,14]. This type of method uses adversarial examples that float on the decision surface during the expansion of the training set, which improves the generalization ability of the model to space the data using high-intensity AT [15,16]. However, these methods usually require huge computing resources and time, and the adversarial trained model can still be attacked by novel adversarial examples. In addition, there is a defense method that resists adversarial attacks by modifying the model structure. Such methods usually require adding additional network layers or regularization terms to the deployed model and then retraining it, which may not be suitable in practical application scenarios.

The above two types of methods are designed with the goal of enhancing the generalization ability of the DNN model itself. In addition, there are some methods to reduce the harmfulness of adversarial noise by modifying the input RSI, which are called adversarial preprocessing (AP). In early exploration, some studies used traditional enhancement operations (e.g., noise addition, grayscale processing, and dithering) to change the visual characteristics of the image in order to blur out the adversarial noise. However, new noises or style differences may bring new challenges to deep learning models and may easily destroy the spatial features and spectral information of RSIs. Later, Gu et al. [17] used adversarial examples to train an additional denoising encoder and achieve certain results. Some studies trained advanced generative models (e.g., generative adversarial network, the energy model, and the diffusion model) to learn the distribution of data spaces, which they then used to destroy the structure of the adversarial noise.

In addition, through the extensive research and continuous improvement of self-supervised representation learning, researchers have found that this technology can provide new ideas for the research on adversarial defense. Hendrycks et al. [18] trained a supervised learning-based target model and a self-supervised learning-based auxiliary network, which provide stronger regularization for adversarial training strategies. Kim et al. [19] proposed a label-independent adversarial attack method and trained the model in the form of self-supervised adversarial learning to maximize the similarity between the enhancement of the input sample and its adversarial noise. Wu et al. [20] used the self-supervised representation to defend against adversarial attacks and designed a layer-wise noise-to-signal ratio to quantify and measure the effectiveness of the self-supervised model in weakening the adversarial noise layer-by-layer. He et al. [21] used a self-supervised learning model to learn feature representations and predicted the labels of the input data; the authors then detected adversarial examples and their enhanced versions based on representation similarity and label consistency. These methods show that self-supervised representation learning has good application prospects in the field of adversarial defense. Nevertheless, there is no research on adversarial denoising at the latent space level.

In order to improve the robustness of RSI scene classification models, we propose an adversarial denoising method based on latent representation guidance. This method takes full advantage of the label-independence of self-supervised representation learning, only uses clean data to train variational autoencoders (VAEs) [22] in the training phase, and does not require any form of adversarial examples and target models. After that, a well-trained model can build a distribution that is determined by latent representations for each input sample. In the iterative denoising phase, we first use normalized mutual information (NMI) to evaluate the reconstructed image and then update the latent distribution according to the reconstruction loss, thereby achieving the purpose of adjusting the latent space distribution. Experiments on RSI scene classification datasets show that the proposed method is effective in defending from adversarial noise. Furthermore, our method achieves better defensive performance compared with state-of-the-art adversarial defense methods in the field of computer vision.

In summary, we provide the following contributions:

- We introduce self-supervised representation learning into the study of adversarial defense methods and design an adversarial denoising method. Because only clean data are used in the training phase, the proposed method is label-independent and model-independent, which is beneficial for improving the model’s defense ability against unknown adversarial noise.
- At test time, we use NMI to measure the quality of the reconstructed image and iteratively update its latent representation. Because the adversarial denoising operation is indirectly completed in the latent space, the proposed method has less impact on the quality and spatial information of images.
- We conduct attack and defense tests on various architectures of RSI scene classification datasets. The results show that the proposed method can effectively reduce the impact of adversarial noise on the model and protect the highly vulnerable RSI scene classification models in the real world.
- To test the performance of our method, we chose state-of-the-art adversarial defense methods in the field of computer vision for comparative experiments, and the results show that the proposed method exhibits a competitive defense performance. Furthermore, the proposed method can be combined with other adversarial defense methods as an additional plugin.

## 2. Related Works

In this section, we first briefly introduce the adversarial attack methods that are involved in our experiments and then review the classic adversarial defense methods; finally, we introduce the principle and development of VAEs.

### 2.1. Adversarial Attacks

Although physical adversarial attacks are not a threat in most remote sensing applications, the ultimate goal of studying adversarial attacks is to increase models’ resilience to overfitting and increase their generalization ability to complex data spaces. Most of the existing adversarial attack methods used in the field of remote sensing extend from the research results in the field of computer vision, such as the fast gradient sign method (FGSM) [4], projected gradient descent (PGD) method [16], Carlini and Wagner (C&W) method [23], DeepFool method [24], AutoAttack method [25], and backward pass differentiable approximation based on expectation over transformation (BPDA+EOT) method [26].

The FGSM method is a gradient-based adversarial attack method; it generates adversarial examples by computing the gradient of the model to the input data and by adjusting the input data according to the direction of the gradient. It is a fast and relatively simple adversarial attack method. The PGD method is an iterative gradient descent adversarial attack method; it generates adversarial examples by applying FGSM on the input data in multiple iterations. At each iteration, PGD will slightly perturb the input data within a certain range to increase the effect of the attack. The C&W method is an optimization-based adversarial attack method; by performing optimizations in the input space, it aims to find adversarial examples that maximize the objective function. This method usually has a high attack success rate but has a high computational cost. The DeepFool method is an iterative linearization adversarial attack method; it uses a linear approximation in the input space to find the direction of the smallest perturbation and slightly perturbs the input data in this direction. DeepFool aims to minimize the magnitude of the perturbation. The AutoAttack method is a comprehensive adversarial attack evaluation framework that integrates a variety of adversarial attack methods; in addition to the above four common methods, it also includes the methods of square attack, boundary attack, etc. It aims to provide users with a one-stop adversarial attack evaluation tool, which can help researchers more fully understand the weaknesses and vulnerabilities of models. The BPDA+EOT method is an adversarial attack method based on back-propagation; it approximates discrete gradient values by using different transformation functions in the forward and back-propagation stages. This approach can be computationally more efficient and relatively effective for attacking.

### 2.2. Adversarial Defenses

#### 2.2.1. Adversarial Training

AT is considered to be one of the most effective defense strategies in the field of deep learning. Its idea is to supplement adversarial examples into the training set as outlier samples that are close to the decision surface so that the model can explore the decision space more comprehensively and capture complex decision boundaries. The higher the quality of adversarial examples during AT, the better the generalization of the model. The following are several classic AT methods. Xu et al. [27] introduced the FGSM-AT model into the RSI scene classification application, which effectively improved the adversarial robustness of the target model. Li et al. [28] used the PGD-AT model to enhance the synthetic aperture radar interpretable image recognition model. Zhang et al. [29] proposed an AT method that satisfies the Lipschitz continuity constraints, which was named TRADES. The model is trained by generating adversarial examples and minimizing the adversarial loss, which improves the robustness and generalization of the model. Cheng et al. [30] introduced the generative adversarial network (GAN) into the AT framework to model the distribution of adversarial noise, aiming to use the pattern discovery ability of GAN to explore unknown types of adversarial noise. However, the authors hoped to explore unknown adversarial noise through several known adversarial attacks, which requires a more detailed demonstration. In order to protect the highly vulnerable salient object detection model, Sun et al. [31] proposed a remote sensing image defense framework based on an adversarial cloud, which can be easily added to the deployed object detection application. Although these methods have achieved good performance, the computational complexity of AT is relatively large, and problems such as catastrophic overfitting may be encountered.

#### 2.2.2. Adversarial Preprocessing

Adversarial preprocessing is the operation of detecting or purifying the data to be tested with the aim of eliminating or modifying samples that contain adversarial noise so as to ensure the accuracy of the model to the greatest extent [32]. Adversarial detection [33] methods can be divided into two categories: feature-based methods and energy-based methods. Feature-based methods mainly judge whether a sample is an adversarial sample by analyzing the features or attributes of the input sample. For example, Li et al. [34] first fused the features output by the first and second fully connected layers of the target model and then used the support vector machine model to find a hyperplane that could separate positive and negative samples, which was performed to realize the detection of adversarial examples. Chen et al. [35] first used the feedback results of the target model for positive and negative samples to additionally train a lightweight classifier; the authors then obtained the confidence threshold of each category according to the decision boundary, which is a soft threshold for detecting adversarial examples. Energy-based methods judge whether a sample is an adversarial sample by calculating the energy or abnormality of the input sample. Zhang et al. [36] proposed an energy-based adversarial detector that uses energy regularization to fine-tune the pretrained model. These methods have a small amount of calculation and are easy to deploy, but have a great impact on the standard accuracy of the target model.

Research on purification methods is more diverse. Researchers treat adversarial noise as ordinary noise and adopt traditional denoising methods to deal with it. For example, Tabacof et al. [37] studied the impact of Gaussian noise with different intensities and distributions on adversarial examples. Raff et al. [38] randomly combined several weak transformation methods, including color precision reduction, JPEG noise, swirl, and FFT perturbations, to destroy the structure of adversarial noise. However, these two methods have a certain impact on the quality of the image and accuracy of the model. Gu et al. [17] first added ordinary noise to adversarial examples and then used a denoising autoencoder to remove adversarial noise. In a later study, the purification framework proposed by Meng et al. [39] could gradually adapt adversarial examples to real data manifolds. Moreover, Liao et al. [40] designed a denoising method based on high-level representation guidance. Xu et al. [41] proposed a denoising network that is guided by the scene classification task that transforms adversarial examples into images that are similar to the corresponding clean data based on the feedback from a target model. It has good performance with respect to removing adversarial noise and improving model robustness. These methods usually require a certain amount of adversarial examples as prior experience, which makes it difficult to deal with unknown adversarial noise. To get rid of the reliance on adversarial examples, Yang et al. [42] designed a denoising method based on the destruction–reconstruction mode called ME-Net by using matrix estimation. Shi et al. [43] proposed a self-supervised online adversarial purification (SOAP) strategy. Similarly, Xu et al. [44] adopted a mode of co-training the target network and additional distillation network, designed label-independent, instance-wise adversarial attack methods, and conducted adversarial training. In addition, Hill et al. [45] introduced an energy-based model (EBM) into the adversarial defense method, and Yoon et al. [46] improved the EBM model by using the denoising score matching (DSM) method. However, EBM relies on huge sampling and calculations; the diffusion model is time-consuming, which is not suitable for applications that require high real-time performance.

### 2.3. Variational Autoencoders

VAEs [47,48] usually consist of an encoder and a decoder. This method works by adding constraints during the encoding process so that the latent vectors generated by the encoder roughly follow a standard normal distribution. Specifically, given a true distribution $\tilde{p}\left(x\right)$ of a batch of samples, the form of the generative model is as follows:

(1) $$p\left(x,z\right)=p\left(z\right)\cdot p\left(x\left|z\right.\right),$$

where $p\left(z\right)$ is a prior distribution over latent variables *z* and $p\left(x\left|z\right.\right)$ is a likelihood function or decoder. Variational inference is an approximate inference method where the goal is to approximate the true posterior distribution $p\left(z\left|x\right.\right)$ by a tractable approximate distribution $p\left(z\right)$. Because the true posterior $p\left(z\left|x\right.\right)$ is generally intractable, the generative models are trained using the aid of an approximate posterior distribution or encoder $q\left(z\left|x\right.\right)$. However, in the world of continuous distributions, there are not many distributions that are convenient to sample, and researchers usually choose Gaussian distributions with independent components to construct $q\left(z\left|x\right.\right)$, $p\left(z\right)$, and $p\left(x\left|z\right.\right)$. As such distributions are difficult to fit to complex distributions, the images generated by VAEs are blurry.

To improve the visual quality of the images that are generated by VAEs, there have been many variants. Some change $p\left(x\left|z\right.\right)$ to a more general distribution. For example, Ma et al. [49] introduced VAE-GAN to RSI scene classification applications in the zero-shot setting, wherein the authors used the discriminator to learn a suitable reconstruction quality metric for the VAE. Zhang et al. [50] embedded the texture-guided prior information into the VAE network and then embedded the spatial-wise attention block into the discriminator, which can generate remote sensing images with more realistic texture details. Heydari et al. [51] proposed an IntroVAE-based single image super-resolution method for learning the latent manifold structure of super-resolved images. Meanwhile, the introduction of GAN brought the problem of mode collapse and an unstable training gradient. Cardenas et al. [52] relied on powerful autoregressive priors of the VQ-VAE to learn the spatial consistency and semantic consistency of images with complex textures. Du et al. [53] proposed a CVAE that can provide a suitable prior probability distribution for latent variables by optimizing the Kullback–Leibler divergence between the conditional prior and approximate posterior probability distribution. Vahdat et al. [22] proposed Nouveau VAE (NVAE) based on multi-scale architecture, which improved the quality of image generation.

## 3. Methodology

The architecture of the classical VAE consists of two main components: an encoder and a decoder. The encoder can capture the basic features of the input data and map it to a compact cluster of latent space, namely the multivariate Gaussian distribution. In particular, to sample from the latent space in a differentiable way, VAEs employ reparameterization techniques to shift the problem into a more general distribution. Meanwhile, the decoder generates new data samples that are similar to the original data based on the sampled latent representation. These representations contain most of the important information that is needed by the data. Analyzing latent representations provides insight into the patterns or structural similarities between data.

When training the VAE, minimize the reconstruction error to ensure that the generated new data are similar to the distribution of the original data and optimize the relative entropy of the latent vector to learn a compact and smooth latent representation. After properly training with clean data, the encoder’s mapping to normal data deviates from its mapping to abnormal data; that is, the latent distribution that is obtained by encoding clean samples and adversarial examples is different.

To fix this “bias”, we adjust the latent distribution during the denoising phase by iteratively updating the latent features. In addition, because the pictures generated by the VAE are usually blurry and cannot meet the needs of practical applications, we chose the derived NVAE [22] model as the engineering implementation.

**Figure 1:**
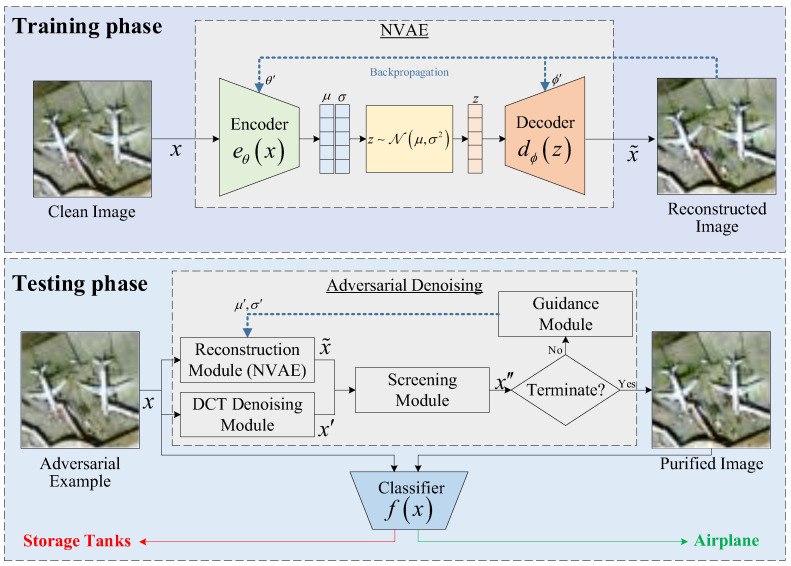
The framework of the adversarial denoising model based on latent representation guidance.

Figure 1 shows the framework of the proposed adversarial denoising model based on the latent representation guidance. It mainly consists of two phases: training of a self-supervised generative model and adversarial denoising at the latent space level. In the training phase, we use a clean dataset to train a deep hierarchical variational autoencoder called NVAE; once trained, its encoder is able to build a latent Gaussian distribution for any input in the sample space. There are a large number of normal samples and a small number of abnormal samples gathered in this distribution. Then, NVAE’s decoder is able to generate a new sample that is similar to the input sample based on the latent representation sampled from the latent distribution. Because NVAE is good at capturing the basic features of input data, some meaningless but adversarial features will be filtered out after the encoding and decoding operations. In the denoising phase, we use NVAE as the reconstruction module, take the DCT denoised image as a reference, and use NMI to evaluate the usability of the reconstructed image for the current iteration. After this step, the latent representation is iteratively updated according to the reconstruction loss, thereby changing the latent distribution of the original sample. After repeated adjustments and sampling and filtering operations, the structure of the adversarial noise is gradually destroyed. This is the specific design idea of the adversarial denoising method.

### 3.1. Training Phase

**Figure 2:**
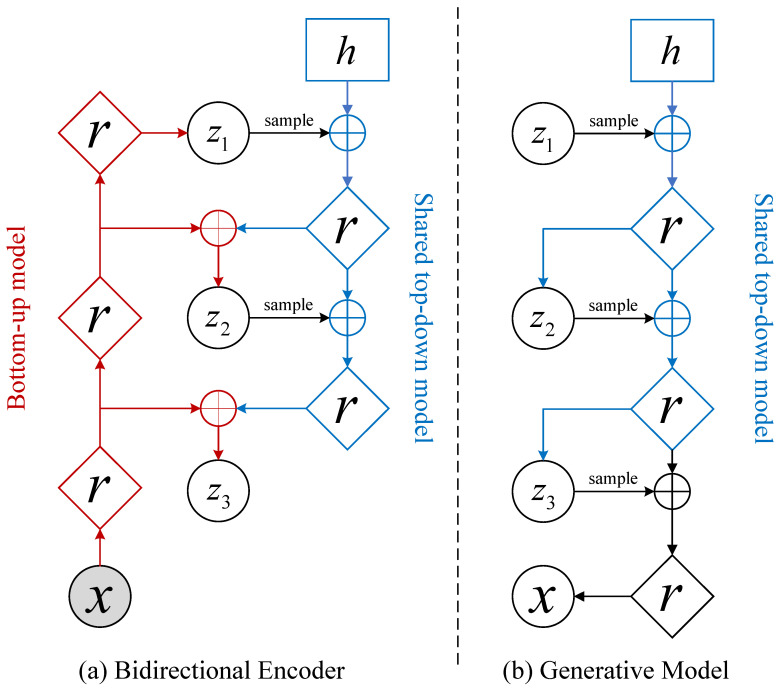
An illustration of the bidirectional encoder and generative model in NVAE.

In this paper, we choose the deep hierarchical NVAE model. We focus on the orthogonal direction of neural architectures for hierarchical VAEs, divide the latent variables into vector groups $z=\left\{z_{1},z_{2},\ldots ,z_{L}\right\}$, and enhance the prior distribution $p\left(z\right)$ and the approximate posterior distribution $q\left(z\left|x\right.\right)$ using autoregressive models. The prior is represented by $p\left(z\right)=\prod_{l=1}^{L}p\left(z_{l}|z_{<l}\right)$ and the approximate posterior is represented by $q\left(z\left|x\right.\right)=\prod_{l=1}^{L}q\left(z_{l}\left|z_{<l}\right.,x\right)$. Then, we use multi-scale architectures and depth-wise separable convolutions to design a bidirectional encoder and a generative model, as shown in Figure 2.

For the input sample *x*, the encoder performs multi-level encoding on the sample *x* and obtains a top-level feature vector $z_{1}$. Then, the encoding vector $z_{l}$($l\in \left[1,L\right)$) of the current layer is sampled to calculate the feature vector $z_{l+1}$ of the next layer. Finally, the encoder obtains a set $Z=\left\{z_{1},z_{2},\ldots ,z_{L}\right\}$ of latent variables that are mutually disjoint, where L is the number of groups. For the lowest-level feature $z_{L}$, there is an approximate posterior distribution of the following form:

(2) $$q_{\Phi }\left(z^{L}|x\right)=\int q_{\phi^{L}}\left(z^{L}|z^{L-1}\right)\cdots q_{\phi^{1}}\left(z^{1}|x\right)dz^{1}\cdots dz^{L-1},$$

where $\Phi =\left\{\phi^{1},\ldots ,\phi^{L}\right\}$.

Because each conditional in the prior $p\left(z_{l}\left|z_{<l}\right.\right)$ and $q\left(z_{l}\left|z_{<l}\right.,x\right)$ follow factorial normal distributions, we assume that $\mu =\left\{m_{1},m_{2},\ldots ,m_{L}\right\}$ and $\sigma =\left\{n_{1},n_{2},\ldots ,n_{L}\right\}$ are the representations of the input sample in the latent space. Additionally, *Z* is sampled from a normal distribution that is determined by μ and σ.

The decoder first calculates latent variables $z_{1},z_{2},\ldots ,z_{L}$ from top to bottom according to the μ and σ output by the encoder. Then, the top-level latent features $z_{1}$ are sampled. The feature combination is performed on the sample with the trainable parameter *h* to obtain a deterministic feature map. For other layers, the decoder combines the deterministic feature map output using the upper layer with the sample taken from $z_{l}$($l\in \left[1,L\right)$) to obtain the deterministic feature map of the current layer. When decoding to the bottom layer, the decoder outputs a new sample that has some correlation with *x*.

When training NVAE, for each sample, we wish to maximize its variational lower bound on $\log p\left(x\right)$ as:

(3) $$L\left(\Phi ,\theta ;x\right)=E_{q_{\Phi }\left(z|x\right)}\left[\log\frac{p_{\theta }\left(x,z\right)}{q_{\Phi }\left(z|x\right)}\right]$$

Given $p_{\theta }\left(x,z^{L}\right)=p_{\theta }\left(x|z^{L}\right)p\left(z^{L}\right)$, we can obtain the following inequality according to the lemma provided by Im et al. [54]:

(4) $$\begin{array}{cc} \log p_{\theta }\left(x\right) & \geq E_{q_{\Phi }\left(z^{L}|x\right)}\left[\log\frac{p_{\theta }\left(x,z^{L}\right)}{\prod_{l=0}^{L-1}q_{\phi^{i}}\left(z^{l+1}|z^{l}\right)}\right]\\ & \geq E_{q_{\Phi }\left(z^{L}|x\right)}\left[\log\frac{p_{\theta }\left(x,z^{L}\right)}{q_{\Phi }\left(z^{L}|x\right)}\right] \end{array}\;$$

The above inequality illustrates that the multi-scale architecture provides a tighter variational lower bound. Such a design can better fit complex continuous distributions. When the NVAE model is properly trained, the sample $\tilde{x}$ output by the decoder will be similar to the initial sample *x*.

### 3.2. Testing Phase

As shown in Figure 1, the test time adversarial denoising model consists of four parts: the reconstruction module, discrete cosine transform (DCT) denoising module, screening module, and latent representation guidance module. When an NVAE model is properly trained, it is able to create a mapping from the input space to the latent space. Because clean samples and adversarial examples have different representations in the latent space, we iteratively update the features of the latent space to gradually alleviate the impact of adversarial noise. Therefore, the latent representation guidance module embodies the core design concept of this method.

Next, we introduce the four modules of the adversarial denoising model sequentially. An input sample *x* is first fed into both the reconstruction module and the DCT denoising module.

#### 3.2.1. Reconstruction Module

In the reconstruction module, the well-trained NVAE model encodes the input *x* to obtain a latent Gaussian distribution as determined by the latent representations $\mu_{0}$ and $\sigma_{0}$. In this distribution, samples with relatively less adversarial noise are densely clustered. Because VAE uses variational inference methods to approximate the posterior probability distribution, the approximation of the posterior distribution may not be accurate. To this end, we default to a deterministic sampling of the latent distribution to ensure the fidelity of the generated data. Then, the decoder generates a reconstructed sample ${\tilde{x}}_{1}$ based on the sampled latent representation. It is worth noting that for the subsequent *i*-th (i>1) iteration, NVAE no longer uses the encoder and only uses $\mu_{i-1}$ and $\sigma_{i-1}$ to determine the reconstruction sample ${\tilde{x}}_{i}$.

Because NVAE is good at capturing the basic features of input data, some meaningless but adversarial features will be filtered out after the encoding and decoding operations.

#### 3.2.2. DCT Denoising Module

In the DCT denoising module, we first perform DCT on *x* to obtain the frequency domain representation $x^{\prime }$ then remove the high-frequency contents to reduce the impact of adversarial perturbations; finally, we obtain the denoised sample $x^{\prime }$ through the inverse discrete cosine transform (IDCT).

DCT denoising has certain advantages in energy concentration, compressibility, reversibility, and applicability. In this paper, the DCT denoised image has the following two uses: one use is as a reference in the screening module to evaluate the usability of the reconstructed image generated by NVAE, and the other is to participate in the calculation of MSE loss in the latent representation guidance module. First, when evaluating the usability of the reconstructed image, if the abnormal sample is used as the reference, using NMI to evaluate the similarity between the reconstructed image and the reference image will make it difficult to make the reconstructed image cleaner. Second, MSE is a pixel-level evaluation metric that is used to measure the difference between images. The model and user cannot predict whether the input sample is adversarial. If the abnormal sample is used to guide the update of latent representation when calculating the reconstruction loss, it will be difficult for the latent distribution to be better calibrated. For this reason, it is more conducive to the overall effect of our method to select traditional denoised images with relatively less adversarial noise as a reference instead of unknown input samples.

#### 3.2.3. Screening Module

After obtaining ${\tilde{x}}_{i}$ and $x^{\prime }$, we use the screening module to decide whether to accept the reconstructed sample ${\tilde{x}}_{i}$ that is output by NVAE. ${\tilde{x}}_{i}$ and $x^{\prime }$ are regarded as clustering results, where each pixel or feature vector can be regarded as a sample. We choose NMI as the image quality evaluation index to compare the similarity between these two clustering results. A higher NMI value indicates that the clustering results of two images are more similar, while a lower NMI value indicates that the clustering results are less similar. In contrast to peak signal-to-noise ratio (PSNR) and Structural Similarity (SSIM), NMI comprehensively considers the structural similarity of images and consistency of clustering results, is robust to nonlinear transformations such as data compression and adversarial attacks, and can better evaluate the obtained images.

Specifically, we first initialize the recording of the NMI value to zero, i.e., $NMI_{0}=0$. For the *i*-th iteration, we calculate the NMI value between ${\tilde{x}}_{i}$ and $x^{\prime }$ using the following formula:

(5) $$NMI_{i}\left({\tilde{x}}_{i},x^{\prime }\right)=\frac{2\times \left(H\left({\tilde{x}}_{i}\right)-H\left({\tilde{x}}_{i}|x^{\prime }\right)\right)}{H\left({\tilde{x}}_{i}\right)+H\left(x^{\prime }\right)},$$

where $i\in \left[1,I_{\max}\right]$, $I_{\max}$ is the maximum number of denoising iterations and $H\left(\cdot \right)$ is the cross-entropy method. Then, the value is compared with the recording $NMI_{i-1}$ from the previous iteration. If $NMI_{i}>NMI_{i-1}$, we use ${\tilde{x}}_{i}$ as the purified sample ${x_{i}}^{\prime \prime }$ and update the recording of the NMI value. Otherwise, we use ${x_{i-1}}^{\prime \prime }$ from the previous iteration as the purified sample for this iteration and keep the recording of NMI unchanged, i.e., $NMI_{i}=NMI_{i-1}$.

#### 3.2.4. Latent Representation Guidance Module

Next, we compute the mean square error (MSE) loss $L_{cal}$ of the purified sample ${x_{i}}^{\prime \prime }$ and the denoised sample $x^{\prime }$ as the guidance loss of the adversarial denoising model.

(6) $$L_{cal}=\frac{1}{N}{\left({x_{i}}^{\prime \prime },x^{\prime }\right)}^{2}$$

After that, we keep the weights of the NVAE model unchanged and use $L_{cal}$ to update the feature representations $\mu_{i}$ and $\sigma_{i}$ of the latent space during back-propagation, as shown in Equations (7) and (8). In particular, such a guidance operation updates the feature representations $m_{l}$ and $n_{l}$ of each layer, where $l\in \left[1,L\right)$. Whenever we pass $\mu_{i}$ and $\sigma_{i}$ into the decoder of NVAE, a calibration of the latent distribution is completed.

(7) $$\mu_{i}=\mu_{i-1}-\frac{\partial L_{cal}}{\partial \mu_{i-1}}$$

(8) $$\sigma_{i}=\sigma_{i-1}-\frac{\partial L_{cal}}{\partial \sigma_{i-1}}$$

In this way, the latent representation is iteratively updated, thereby changing the latent distribution of the original samples. After repeated adjustments and sampling and filtering operations, the structure of the perturbations is gradually destroyed, and the reconstructed samples are less deceptive to the target model.

## 4. Experimental Evaluation

### 4.1. Datasets and Network Architectures

In this section, we first test the effectiveness of the proposed method for defending against adversarial noise on the UC Merced (UCM) land use dataset [55]. Then, we compare our method with baseline adversarial defense methods in the field of computer vision on the CIFAR-10 dataset [56].

UCM is a remote sensing image dataset for land-use classification and recognition tasks; it covers 21 different land use categories, such as urban areas, farmlands, forests, grasslands, lakes, rivers, highways, etc. Each category consists of 100 high-resolution aerial images with a resolution of 256 × 256 pixels. We randomly split the UCM into the training set and test set at a ratio of 8:2. Therefore, the training set and test set contain 1680 and 420 remote sensing images, respectively. To this end, the experimental results on the UCM dataset are based on the average accuracy obtained from five independent experiments.

CIFAR-10 is a computer vision dataset for image classification tasks. It covers 10 different object classes, i.e., airplanes, cars, birds, cats, deer, dogs, frogs, horses, boats, and trucks. These images have certain complexity and variability, which is challenging for the training and evaluation of deep learning models. Each category consists of 6000 images with a resolution of 32 × 32 pixels.

### 4.2. Experimental Settings

When training target models to be attacked, we first perform preprocessing operations such as random cropping, scaling, and horizontal flipping on the training set and then perform 200-epoch training. During this phase, a cosine annealing learning rate adjustment algorithm with a maximum period of 200 and batch normalization techniques are used to speed up the optimization of the model.

We compare the proposed method against widely-used AT and AP methods on a variety of $l_{\infty }$- and $l_{2}$-bounded attacks: FGSM, PGD, C&W, DeepFool, AutoAttack, and BPDA+EOT. For UCM, both FGSM, PGD, and AutoAttack are $l_{\infty }$ bounded with ε=0.01, and the PGD runs 20 iterations with a step size of 0.002; C&W and DeepFool are $l_{2}$-bounded with ε=2. For CIFAR-10, both FGSM, PGD, AutoAttack, and BPDA+EOT are $l_{\infty }$-bounded with ε=8/255, and the PGD runs 20 iterations with a step size of 2/255; C&W and DeepFool are $l_{2}$-bounded with ε=2. In addition, the number of parallel samples used for the EOT attack is 15, and the number of parallel purification trials for verifying successful attacks with EOT defense is set to 150.

Next, we introduce the experimental settings of the proposed algorithm. When training NVAE, the main parameter settings are listed in Table 1. In the denoising phase, we choose NMI as the image quality assessment index in the screening module and set the number of iterations to 200.

### 4.3. Effectiveness on Remote Sensing Dataset

We conduct experiments on four classic image classification models, i.e., LeNet with a standard accuracy of 70.00%, VGG16 with a standard accuracy of 77.14%, AlexNet with a standard accuracy of 77.86%, and ResNet-18 with a standard accuracy of 85.95%. The accuracy of the target model is equal to the ratio between the number of recognition results that are consistent with the true label and the total number of the test images. Table 2 shows the robust accuracy of the adversarial denoising method against different adversarial attack algorithms on the UCM dataset.

**Figure 3:**
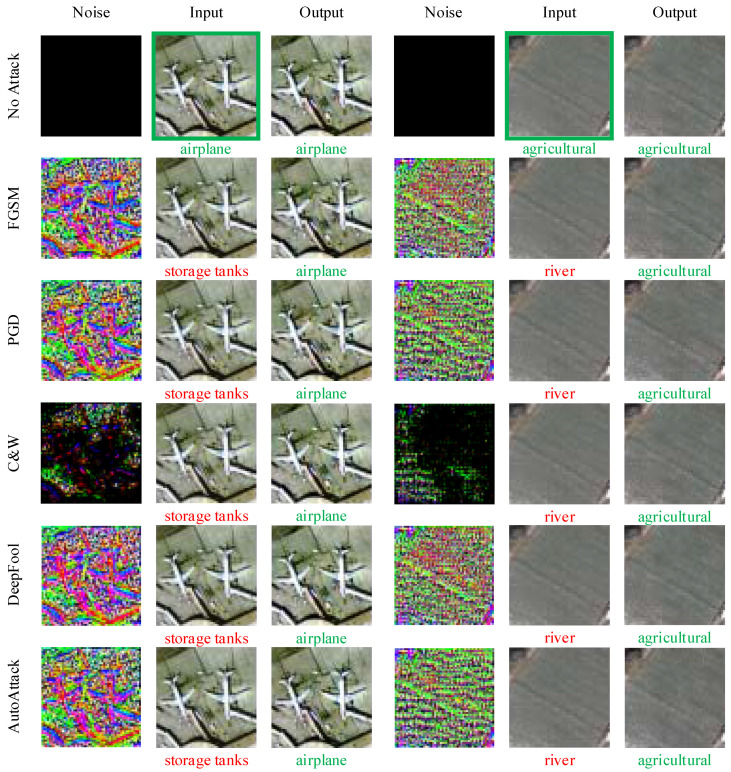
Examples of adversarial attacks and adversarial denoising.

We can see that when the input is clean data, the standard accuracy of the target model on the reconstructed data is slightly reduced. This is a common problem that is often associated with adversarial defense methods. Adversarial purification methods may lead to the loss of meaningful features, and adversarial training methods may lead to the overfitting of adversarial examples. Meanwhile, when the input is an adversarial example, the classification accuracy of the target model on the reconstructed data is significantly improved. In Figure 3, we show examples of the adversarial noise that is produced by these attack methods, the generated adversarial examples, and the reconstructed images after denoising.

#### 4.3.1. Effectiveness across Different Models on CIFAR-10

**Figure 4:**
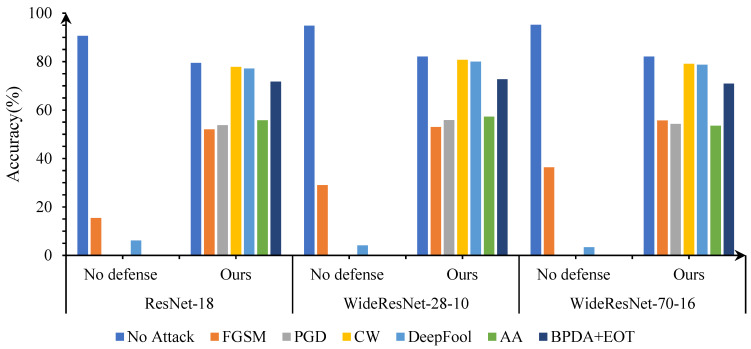
The defense performance of the proposed method against different adversarial attacks on CIFAR-10.

In this part, we first use classic adversarial attack algorithms to attack advanced object classification models in the CIFAR-10 dataset. Then, we test the effectiveness of the proposed adversarial denoising method for defending against adversarial attacks, as shown in Figure 4.

The following points can be seen from the Figure 4: first, the proposed method has good robust accuracy, especially for $l_{2}$-bounded attacks such as C&W and DeepFool; second, when tested using clean data, the model still maintains a good standard accuracy; third, as the complexity of the model increases, the success rate of adversarial attacks decreases, but the robustness of our method remains good.

#### 4.3.2. Comparison with Adversarial Purification Methods

In this part, we test the adversarial attacks and adversarial purification methods on CIFAR-10 using WideResNet-28 as the target model, as shown in Table 3. We mark the best performance for each attack by an underlined and bold **value** and the second best by a bold **value**.

The adversarial attack algorithms that we chose were BPDA+EOT and AutoAttack. BPDA+EOT is the main attack method for evaluating adversarial purification algorithms [45,46]. Adversarial purification methods include ME-Net, EBM, DSM, and SOAP. For experiments using ME-Net, we set the masking probability to $\left[0.4,0.6\right]$ and used the nuclear norm minimization method for matrix estimation. For experimental results using EBM and DSM, we borrowed the results from the paper by Yoon et al.; the authors introduced Langevin dynamics (LD) into the sampling process when implementing these two methods. For experiments using SOAP, we first chose label consistency as self-supervised signals and used the auxiliary loss to set the budget of purification. Then, we tested SOAP with a budget of five iterations and a step size of 4/255.

These methods only require clean data to participate in training. The performance comparison of these method are shown in Table 3. We can see that ME-Net has the lowest impact on the standard accuracy of the target model. This is because this method performs a large number of masking and reconstruction operations on the clean dataset and then conducts large-scale generalization training on the model. However, ME-Net is weak against BPDA+EOT and AutoAttack. Next, SOAP also has less impact on the standard accuracy of the model, and it achieves an accuracy of 87.00% on the clean dataset. This is because SOAP adopts a design scheme of joint training of the classification model and auxiliary model, which reduces the impact on the classification model. Meanwhile, both the EBM and DSM models have greatly improved the robustness against previous methods. However, as both methods have optimization or a large number of sampling loops in their defense process, it is difficult to test their defense capabilities against AutoAttack. In comparative experiments, the proposed method allows the target model to have the best defense performance against adversarial examples. After adversarial denoising, the robust accuracy of the model is as high as 72.68% for adversarial examples that are generated by BPDA+EOT and 57.31% for adversarial samples that are generated by AutoAttack.

#### 4.3.3. Compatibility with Prior Arts

**Figure 5:**
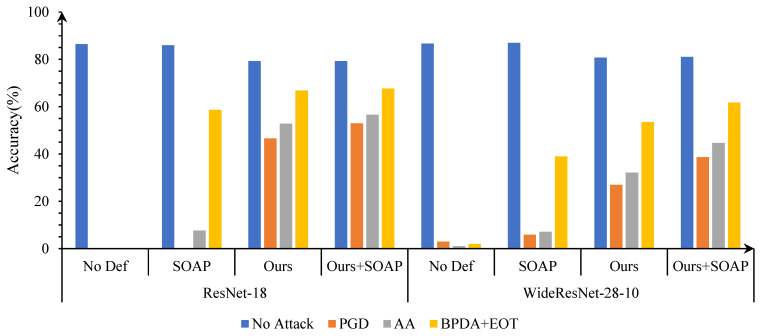
Compatibility testing of the proposed algorithm with SOAP.

Because SOAP is trained on classification tasks and self-supervised tasks, we separately tested the performance of combining the proposed adversarial cleaner as a plugin with the SOAP framework, as shown in Figure 5. Obviously, after multi-task learning and purification, the robust accuracy of the model is greatly improved. In particular, when the proposed denoising method is combined with SOAP, better defense performance is achieved in various attack tests.

In addition, we tested a classic AT method, TRADES, and conducted experiments that combined the proposed denoising method with TRADES. When training TRADES, we set the step size for perturbations to 0.007, the weight decay to $2\times 10^{-4}$, the number of perturbation iterations for PGD to 10, and a trade-off regularization parameter β to 6.0. A 10-widen WideResNet-34 was chosen as the architecture of the image classification model.

**Figure 6:**
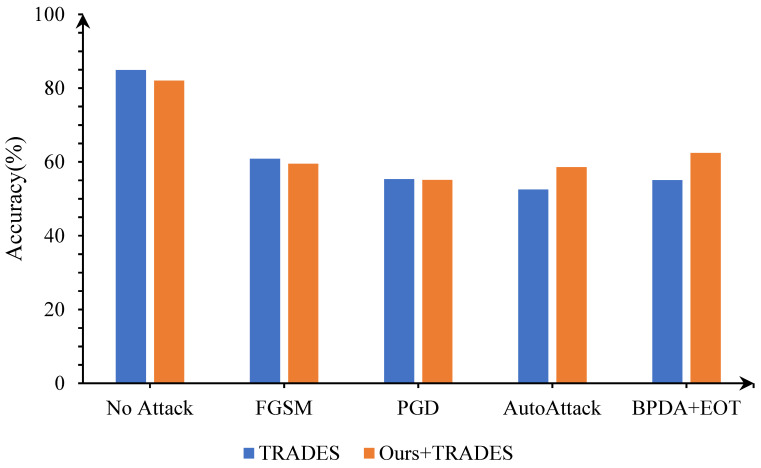
Compatibility testing of the proposed algorithm with TRADES.

As shown in Figure 6, when the proposed adversarial denoising model is used as a plug-in with TRADES, the defense strategy inherits the excellent performance of our method against AutoAttack and BPDA+EOT. After purification and adversarial training, the robust accuracy of the model is as high as 58.57% for adversarial samples that are generated by AutoAttack and 62.41% for adversarial samples that are generated by BPDA+EOT.

### 4.4. Ablation on Experimental Settings

Due to the limitation of GPU memory, it is inconvenient for us to use the UCM dataset to test the larger-scale VAE. Therefore, we first used the CIFAR-10 dataset to test the optimal parameter settings of the NVAE part and then used the UCM dataset to test the parameters involved in the proposed denoising method.

#### 4.4.1. Parameters Involved in NVAE

Although Nie et al. have concluded through experiments that NVAE cannot purify adversarial examples [57], we found that it is feasible to use NVAE as a denoising model as long as the appropriate parameters are selected. To this end, we first tested the effect of NVAE on the performance of the adversarial denoising under different parameter settings.

**Figure 7:**
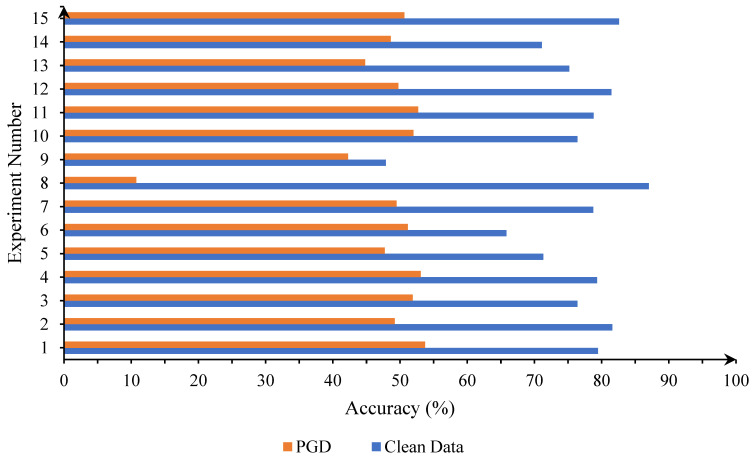
Influence of different parameter settings of NVAE on the defense performance. The part with the pattern filling is the standard accuracy and the part without filling is the robust accuracy.

Table 4 tests the impact of tuning important parameters of NVAE on the defense performance. In this table, Experiment No. 1 completely followed the parameter settings listed in Table 1 without any changes. For the rest of the experiments, we only adjusted a certain parameter setting listed in Table 1 and kept the other parameters unchanged. We considered two metrics to evaluate the performance of the defense approaches: standard accuracy and robust accuracy. The standard accuracy measures the performance of the defense method on a clean test set. The robust accuracy measures the performance on adversarial examples that are generated by different adversarial attack algorithms. In order to facilitate the observation of the experimental results, we drew the data in Table 4 into a histogram, as shown in Figure 7. It can be seen from the figure that the overall performances of the three experiments numbered 1, 4, and 15 are better. We selected the settings of experiment No. 1 with the highest robust accuracy as the configuration of subsequent experiments.

#### 4.4.2. Parameters Involved in Adversarial Denoising

**Figure 8:**
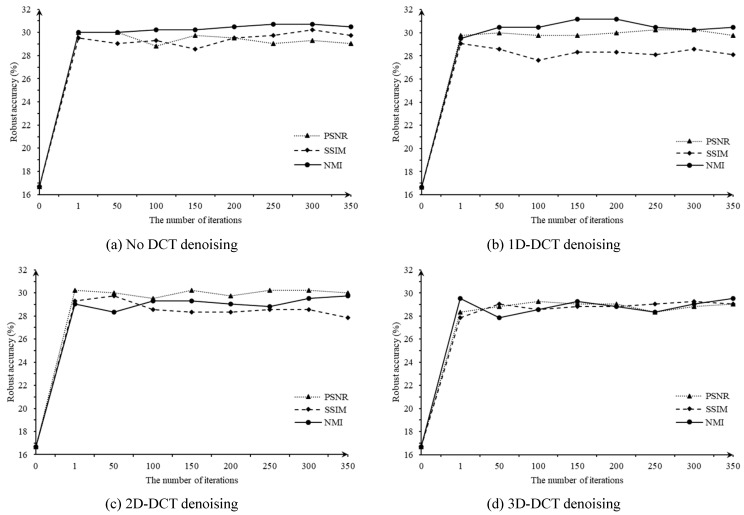
Influence of different image quality assessment and iterations on the proposed algorithm.

After determining the parameters of NVAE, we tested the parameters involved in the proposed adversarial denoising model on the UCM dataset. We tested the influence of the number of iterations, assessment metrics in the screening module, and level of DCT denoising, as shown in Figure 8. Overall, the robust accuracy under different settings in Figure 8b is better. Therefore, in the DCT denoising module, we decided to use 1D-DCT denoising. When using NMI as the image quality assessment in the screening module, the proposed algorithm almost achieves a better performance at different iterations. Finally, it can be seen from Figure 8a,b that when the number of iterations is 200 or 250, the performance of adversarial defense is better. Therefore, we set the number of iterations to 200.

## 5. Discussion

Remote sensing images have the characteristics of high spatial resolution, rich spectral information, and unclear foregrounds and backgrounds. It is difficult for traditional image processing methods to analyze them effectively. Deep learning has the ability to learn complex feature representation and pattern recognition. This technology has achieved remarkable results in RSI analysis tasks, such as classification, semantic segmentation, and the detection of change of land use. However, in the field of remote sensing, factors such as the atmosphere, clouds, noise, and motion artifacts may reduce the quality of RSIs, thereby affecting the analysis of DL models. In particular, the existence of adversarial examples poses a serious threat to the reliability and security of RSIs.

To improve the robustness and reliability of DL models, we proposed an adversarial denoising method based on latent representation guidance for remote sensing image scene classification. Our method does not involve the target model and adversarial examples during the training phase and thus is robust to unknown types of adversarial noise. On the classic remote sensing dataset UCM, we used a variety of mainstream adversarial attack methods to conduct attack and denoising tests. The experimental results show that the proposed adversarial denoising method is effective, especially when defending against L2-bounded attacks such as C&W and DeepFool. Additionally, from the noisy image in the examples shown in Figure 3, it can be found that the adversarial noise produced by the C&W method is relatively concentrated, and it is more suitable for creating physical adversarial examples for RSI. In addition, in order to compare the results with state-of-the-art adversarial defense methods, we also used the computer vision dataset CIFAR-10 for comparative experiments. The experimental results show that the proposed method achieves competitive robust accuracy and can be combined with other adversarial defense methods as a preprocessing plugin.

The idea of the proposed method is to first train a self-supervised generative model and then adjust its latent distribution by updating the latent representation of the data to achieve the purpose of filtering adversarial noise. Within the process, the deep hierarchical variational autoencoder is an important part of our method, which may have the following potential limitations:

- The VAE assumes that the latent space is continuous, which means that samples corresponding to adjacent points in the latent space should also be similar in the data space. This may not be true for data in domains such as natural language or drug molecules.
- The VAE may encounter challenges in sampling and reconstruction when processing high-dimensional data.
- This paper is a study on the robustness of deep learning models, which is suitable for slight adversarial noise removal tasks but not for cloud removal [58] and image deblurring tasks. For example, we tested the robustness of the target model to impulsive noise attacks on the CIFAR-10 dataset. The proposed method reduces the prediction accuracy of the model for these blurred images from 50.10% to 27.45%. Of course, such performance is normal among the existing adversarial defense methods.

Adversarial denoising technology can remove noise and interference in images by learning the noise distribution or characteristics of perturbations, thereby improving the effect and reliability of the RSI analysis. In the future, adversarial denoising technology has broad application prospects in the generalizability and robustness of DL models, super-resolution reconstruction, multi-modal image purification, medical image processing, etc.

## 6. Conclusions

In this paper, we designed an adversarial denoising method for RSI scene classification using a VAE with a multi-scale architecture. During the training phase, we learned the intrinsic features and meaningful representations of the clean data in the form of self-supervised learning. At test time, we first measured the quality of the reconstructed image output from the decoder according to the NMI. Then, this image was reconstructed under the guidance of the latent representations. Several iterations were performed to gradually weaken or eliminate the influence of the adversarial noise. The experimental results show that the proposed method is effective in RSI scene classification tasks and achieves adversarial robustness that is competitive with advanced computer vision adversarial defense methods. In the future, we will continue to seek theoretical innovations in self-supervised learning-based adversarial defense methods within the field of remote sensing.

## Figures and Tables

**Table 1:** A summary of the hyperparameters used in training NVAE.

Hyperparameter	Value
Epoch	200
Batch size	200
Normalizing flows	0
Latent variable scales	1
Groups in each scale	10
Residual cells per group	1
Channels in Z	20
Initial channels in enc./dec.	32
Preprocessing/postprocessing blocks	2
Cells per block	3
Mixture components in dec.	10

Notes: Z represents the set of latent variables.

**Table 2:** The robust accuracy (%) of the proposed method against different adversarial attacks on UCM.

Model	Method	No Attack	FGSM	PGD	C&W	DeepFool	AutoAttack
LeNet	No Def	70.00	19.52	10.24	0.00	0.00	8.57
	Ours	69.76	35.72	31.67	64.05	61.19	39.52
VGG16	No Def	77.14	20.95	6.19	0.00	0.00	4.29
	Ours	76.19	24.05	15.48	52.29	52.38	18.33
AlexNet	No Def	77.86	23.57	10.71	0.00	0.00	3.81
	Ours	76.19	43.33	42.38	72.14	72.62	46.19
ResNet-18	No Def	85.95	28.33	19.76	0.00	0.00	16.67
	Ours	83.33	33.57	26.67	57.38	55.00	31.19

**Table 3:** The performance comparison of different adversarial purification algorithms.

Method	Standard Acc	Robust Acc	
		**BPDA+EOT**	**AutoAttack**
No Defence	90.62	0.00	0.00
Me-net	**87.20**	15.00	**26.30**
EBM+LD	84.12	54.90	-
DSM+LD	86.14	**70.01**	-
SOAP	**87.00**	38.97	7.10
Ours	79.44	**72.68**	**57.31**

Notes: CD and AE represent clean data and adversarial examples, respectively.

**Table 4:** Effects of different parameters in the NVAE model on the performance of the adversarial denoising model.

No	Change	Standard Acc	Robust Acc
1	No changes	79.44	53.73
2	Channels in Z=10	81.57	49.21
3	Channels in Z = 30	76.38	51.88
4	Groups in each scale = 5	79.30	53.08
5	Groups in each scale = 15	71.32	47.72
6	Channels in enc./dec. = 16	65.83	51.17
7	Channels in enc./dec. = 48	78.76	49.47
8	Preprocessing/postprocessing blocks = 1	87.03	10.75
9	Preprocessing/postprocessing blocks = 3	47.88	42.26
10	Cells per block = 2	76.4	52.00
11	Cells per block = 4	78.82	52.70
12	Epoch = 100	81.45	49.74
13	Epoch = 300	75.17	44.79
14	Epoch = 100	71.10	48.61
15	Epoch = 300	82.58	50.65

Notes: Z represents the set of latent variables.

## Data Availability

The UC Merced Land Use dataset and CIFAR-10 dataset are available inside this paper’s References.
